# Linear Augmentation for Stabilizing Stationary Solutions: Potential Pitfalls and Their Application

**DOI:** 10.1371/journal.pone.0142238

**Published:** 2015-11-06

**Authors:** Rajat Karnatak

**Affiliations:** Nonlinear Dynamics and Time Series Analysis Research Group, Max–Planck–Institute for the Physics of Complex Systems, Nöthnitzer Str. 38, 01187 Dresden, Germany; Universitat Pompeu Fabra, SPAIN

## Abstract

Linear augmentation has recently been shown to be effective in targeting desired stationary solutions, suppressing bistablity, in regulating the dynamics of drive response systems and in controlling the dynamics of hidden attractors. The simplicity of the procedure is the main highlight of this scheme but questions related to its general applicability still need to be addressed. Focusing on the issue of targeting stationary solutions, this work demonstrates instances where the scheme fails to stabilize the required solutions and leads to other complicated dynamical scenarios. Examples from conservative as well as dissipative systems are presented in this regard and important applications in dissipative predator—prey systems are discussed, which include preventative measures to avoid potentially catastrophic dynamical transitions in these systems.

## Introduction

Studies on coupled nonlinear systems have explored a wide variety of emergent dynamical phenomena, namely synchronization [1], oscillator suppression [2], multistability [3], hysteresis [4], extreme—events [5, 6] etc. which can be exploited in applications: to model natural phenomena or in regulating the system behavior for instance. Controlling dynamical systems towards a desired behavior is an important research topic in nonlinear sciences [7]. Starting with chaos control [8–11], research in this domain now also extends towards control of multistability [12], patterns and spatio—temporal chaos [13, 14], noisy systems [15, 16], methods of stabilizing unstable stationary solutions [17, 18] etc. A better understanding of these different regulatory aspects has greatly contributed towards development of related novel and highly efficient procedures. Considering noninvasive (without changing the intrinsic system parameters) mechanisms leading to stabilization of stationary solutions, oscillator suppression via coupling nonlinear systems has been discussed extensively in literature (see Refs. [17, 18] for detailed reviews). This suppression is majorly observed as a consequence of parameter heterogeneity between coupled units [19–21], presence of time—delayed [22, 23]/conjugate variables [24] in the coupling function or through dynamic coupling [25], etc.

Recently, linear augmentation has been suggested as another practical alternative leading to oscillator suppression, which is achieved by coupling systems to a linear feedback which simply consists of an exponentially decaying function [26] in the uncoupled state. Interestingly, the coupling structure of linear augmentation is quite reminiscent of indirect or environmental coupling procedures [27–29] which are motivated by observations of collective behaviors in several real world systems, namely, behavior of chemical relaxation oscillators globally coupled through the concentration of chemicals in a common solution [30], dynamics of multi—cell systems where the cells interact through common complex proteins [31], and collective behavior of cold atoms in the presence of a coherent electromagnetic field and atomic recoil [32, 33] for instance. These instances therefore also serve as good examples of systems where linear augmentation can exist naturally. Lately, studies have also effectively used linear augmentation in controlling bistability [34], in controlling the dynamics of drive response systems [35] and in controlling hidden attractors [36].

With respect to stabilizing stationary solutions, Ref. [26] discusses results for an augmented Lorenz oscillator where either the stationary solutions of the Lorenz system (desired) or those of the augmented system could be stabilized by picking an appropriate feedback function. The paper also presents some parameter space plots highlighting the regimes where linear augmentation works in stabilizing the solutions of the original system and where it does not. These results although instructive, are also extremely system specific. At this point one must question the ability of linear augmentation towards stabilizing the desired stationary solutions in a more general sense, namely, for which systems, parameter values and coupling configurations will the scheme work? Since the stability of stationary solutions is determined by the eigenvalues of the augmented system’s Jacobian in the linear approximation, if the eigenvalues are independent of augmentation, or if they remain positive for all values of the augmentation strength, then the procedure will never stabilize these stationary solutions. Furthermore, linear augmentation introduces new stationary solutions in the dynamics which might get stabilized instead of the intended solutions based on similar arguments. In this paper, we will look at some simple examples of linearly augmented systems to highlight that we need to be quite careful before choosing linear augmentation in such applications. These examples illustrate that there could be situations where even picking an appropriate feedback function does not guarantee that the required stationary solutions will be necessarily stabilized; since the mechanism is highly dependent on the intrinsic properties of the oscillators in consideration, the stationary solutions to stabilize, and also on how these systems are coupled to the feedback/ augmented. More importantly, we will also discuss instances where the failures of this procedure can be exploited in meaningful applications, especially in predator—prey systems where we can potentially avoid catastrophic transitions using linear augmentation.

The manuscript is arranged as follows: Sec. Methods briefly introduces the linear augmentation scheme. In Sec. Results, we will look at augmented Harmonic oscillator, and Duffing oscillator as examples of augmented conservative systems followed by augmented Dissipative predator—prey models where the scheme fails to stabilize the desired stationary solutions, and also discuss possible applications for these observations in the latter. Details regarding certain dynamical aspects of harmonic oscillator and Duffing system are provided in Sec. Additional details for completeness. The results and outlook of this work are summarized in Conclusions.

## Methods

### Linear augmentation

General representation of a linearly augmented dynamical system is,

$$\begin{array}{c} \dot{x}=f\left(x\right)+\varepsilon u\\ \dot{u}=-ku-\varepsilon \cdot \left(x-b\right). \end{array}\}$$ (1)

where the column vector $x={\left[x_{1},x_{2},\ldots ,x_{N}\right]}^{T}\in R^{N}$ ([…]^*T*^ corresponds to the transpose) contains the systems variables, and *u* is the augmentation variable. $\varepsilon ={\left[\varepsilon_{1},\varepsilon_{2},\ldots ,\varepsilon_{N}\right]}^{T}\in R^{N}$ is the column vector with information regarding the coupling strength of the interaction between the dynamical variables and *u*; augmentation/coupling term corresponding to the *i* ^*th*^ component *x*_*i*_ is *ε*_*i*_ *u* ∀ *i* = 1, 2, …, *N*, and *ε*_*i*_ = 0 if *x*_*i*_ is not coupled to *u*. $b\in R^{N}$ is an arbitrary vector and *k* is the decay constant [37] which apparently can be used to control the transient time leading to stationary solutions [26]. Vector $b=x^{*}={\left[{x_{1}}^{*},{x_{2}}^{*},\ldots ,{x_{N}}^{*}\right]}^{T}\in R^{N}$ where **x** ^*^ satisfies $\dot{x}{|}_{x=x^{*}}=f(x^{*})=0$ if we want to stabilize a stationary solution **x** ^*^ of the original system. Substituting a value of **b** ≠ **x** ^*^ can stabilize stationary solutions of augmented system for which $\dot{X}={\left[\dot{x_{1}},\dot{x_{2}},\ldots ,\dot{x_{N}},\dot{u}\right]}^{T}\in R^{N+1}=0$. The term *ε*⋅(**x** **−** **b**) gives the dot product of the corresponding column vectors.

In the following, we will look at examples of augmented conservative and dissipative dynamical systems which highlight the limitations of this procedure. The terms *augmentation/augmented* and *coupling/coupled* are used synonymously in the following text.

## Results

Here we will discuss some instances of systems controlled via linear augmentation. We will first look at two examples of conservative systems, namely the harmonic oscillator and conservative Duffing oscillator where linear augmentation is employed to stabilize their stationary solutions.

### Harmonic oscillator

**Fig 1:**
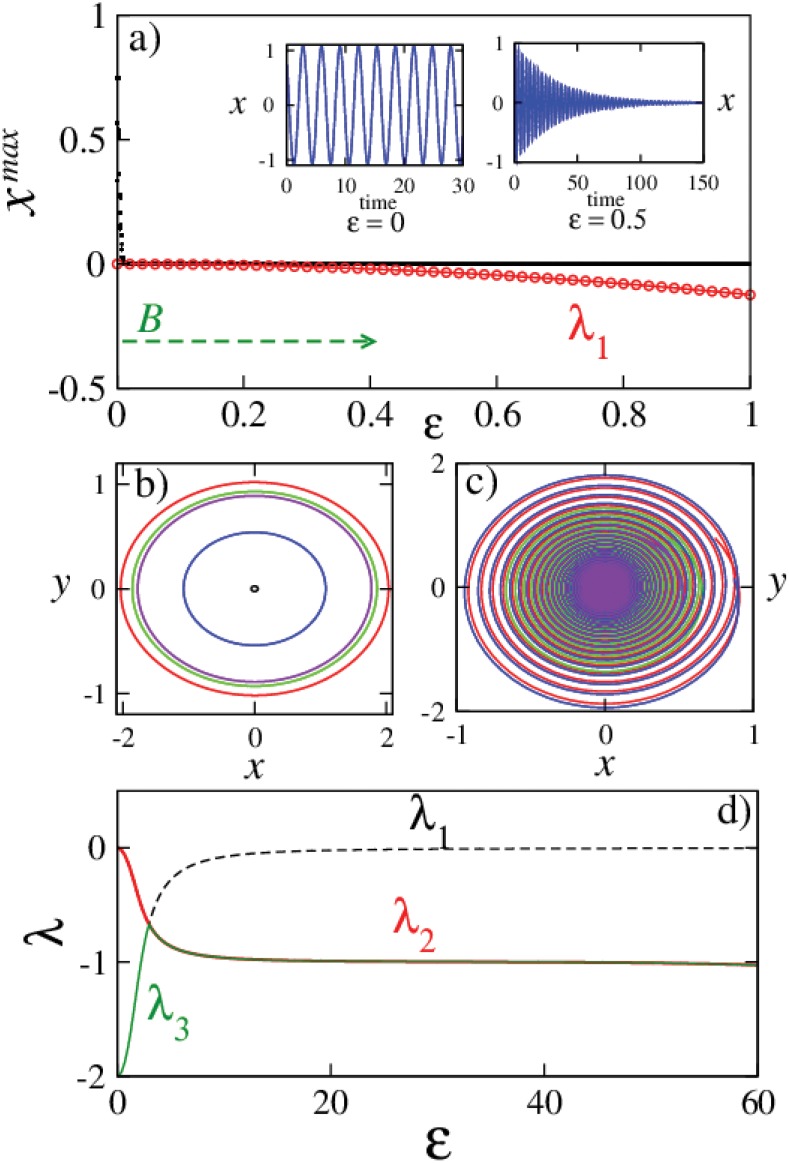
Harmonic oscillator behavior with increasing augmentation strength. a) Bifurcation diagram (black dots), largest eigenvalues (red symbols) and the time series of the *x* variable before and after the stabilization of origin (inset), which occurs for arbitrarily small values of *ε*. *B* marks the stable origin regime which extends to *ε* → ∞. Phase space plots for five different initial conditions, b) for *ε* = 0 (conservative dynamics), and c) for *ε* = 0.5 (dissipative dynamics). d) Shows the variation in the eigenvalues for higher values of augmentation strength *ε*. The other parameter values are fixed at *ω* = 2 and *k* = 2.

Equations describing a partially linearly augmented harmonic oscillator are:

$$\begin{array}{ccc} \dot{x} & = & y+\varepsilon u,\\ \dot{y} & = & -\omega^{2}x,\\ \dot{u} & = & -ku-\varepsilon x, \end{array}$$ (2)

where *ω* is the frequency of the oscillator, *k* is an augmentation parameter, and *ε* is the coupling strength. The first two equations governing the evolution of *x* and *y* correspond to the original harmonic oscillator dynamics of position and momentum respectively. In the absence of augmentation, harmonic oscillator conserves total energy, which stays constant on the ellipses shown in Fig 1b. Each of these ellipses correspond to the systems’ evolution following different initial values of position and momentum, and hence, different conserved total energies. Harmonic oscillator has x* = 0, y* = 0 as the only stationary solution and note that the augmentation term only appears in the rate equation of the position variable *x* at this point. In case of a successful stabilization, the required stationary solution of the full system should be (x*, y*, u*) = (0, 0, 0) (*origin*) where the system effectively decouples from the controller.

The characteristic eigenvalue equation at the origin for this system is,

$$\begin{array}{c} \left(\lambda +k\right)\left(\lambda^{2}+\omega^{2}\right)+\varepsilon^{2}\lambda =0. \end{array}$$ (3)

For *ε* = 0, the eigenvalues for the full system are *λ*_1,2_ = ±*iω* corresponding to the non-hyperbolic stationary solution at the origin, and *λ*_3_ = −*k* corresponds to the decay of the control variable *u*: *u*(*t*) ∝ *exp*(−*kt*) in this case. The other parameter values for the following calculations are fixed at *ω* = 2, and *k* = 2. For the evolution of the augmented system (*ε* > 0), the bifurcation diagram of the system with increasing *ε* values is shown in Fig 1a (black dots). For this calculation, the system is evolved for 10 different initial conditions for each value of *ε* sufficiently for the transients to be discarded. System is then further evolved to capture the possible dynamical regimes by recording all the extrema during the evolution. It is seen that with an increasing *ε*, the system which was conservative for *ε* = 0 becomes dissipative and gets into a stable origin regime even for arbitrarily small values of *ε*. Rewriting Eq 3 as,

$$\begin{array}{c} \lambda^{3}+k\lambda^{2}+\left(\omega^{2}+\varepsilon^{2}\right)\lambda +k\omega^{2}=0, \end{array}$$ (4)

and applying the Routh—Hurwitz criteria (RHC) [38], we can deduce that the roots of this equation are all either negative or have negative real parts (in case of complex roots) ∀ *ε* > 0. Largest eigenvalues of the Jacobian (red symbols) are also plotted along with the bifurcation diagram in Fig 1a which demonstrate the transition from oscillatory to stationary state for any *ε* > 0. Considering the behavior of this system for large *ε*, we can see that the largest eigenvalue *λ* = *λ*_1_ → 0 from Eq 4 in this limit. Since the discriminant (for a general cubic equation *f*(*x*) = *ax* ^3^ + *bx* ^2^ + *cx* + *d*, the discriminant Δ = 18*abcd* − 4*b* ^3^ *d* + *b* ^2^ *c* ^2^ − 4*ac* ^3^ − 27*a* ^2^ *d* ^2^) of the cubic characteristic Eq 4 is negative ∀ *ε* ≥ 0, this implies that the system has one real eigenvalue and a pair of complex conjugate eigenvalues in this range. The negative real part of these complex eigenvalues for large *ε* can therefore be estimated by equating the sum of all eigenvalues to the trace of the Jacobian tr(*J*), giving Re(*λ*_2,3_) = −*k*/2. This further implies that as *ε* → ∞, convergence to the origin gets slower although origin is stable in the entire *ε* > 0 range and any change in stability will only occur as *ε* → ∞ when *λ*_1_ = 0.

Now let us consider a more general case of an augmented harmonic oscillator given by,

$$\begin{array}{ccc} \dot{x} & = & y+\varepsilon_{1}u,\\ \dot{y} & = & -\omega^{2}x+\varepsilon_{2}u,\\ \dot{u} & = & -ku-\varepsilon_{1}x-\varepsilon_{2}y, \end{array}$$ (5)

where the augmentation now appears in the rate equations of both position *x* and momentum *y* with coupling strengths *ε*_1_, *ε*_2_ respectively. The eigenvalue equation in this case is,

$$\begin{array}{c} \left(\lambda +k\right)\left(\lambda^{2}+\omega^{2}\right)+\lambda \left({\varepsilon_{1}}^{2}+{\varepsilon_{2}}^{2}\right)+\varepsilon_{1}\varepsilon_{2}\left(1-\omega^{2}\right)=0. \end{array}$$ (6)

Substituting *ε*_2_ = 0 and *ε*_1_ = *ε* in Eq 5 yields the dynamics of Eq 2. Similarly, for *ε*_1_ = 0 and *ε*_2_ = *ε*, we obtain a case where the system is only coupled in the *y* variable for which the characteristic Eq 6 is exactly identical to Eq 4, and therefore the stability characteristics of the origin are identical and independent of whether the system is augmented in *x* or *y*. In the previous example, we saw a situation where linear augmentation successfully stabilized the origin for the entire range of *ε* > 0. Now considering *ε*_1_ = *ε*_2_ = *ε* (the system is similarly augmented in both variables), in which case Eq 6 gives,

$$\begin{array}{c} \lambda^{3}+k\lambda^{2}+\left(\omega^{2}+2\varepsilon^{2}\right)\lambda +k\omega^{2}+\varepsilon^{2}\left(1-\omega^{2}\right)=0. \end{array}$$ (7)

Using the RHC, it can be checked that this equation will have all negative eigenvalues iff *kω* ^2^ + *ε* ^2^(1 − *ω* ^2^) > 0 which gives a stability regime of 0 < *ε* < ε* ∀ *ω* > 1 where $\varepsilon^{*}=\omega \sqrt{\frac{k}{\omega^{2}-1}}$, and for higher values of *ε*, RHC suggests appearance of positive eigenvalue/eigenvalues. For large *ε*, we can get an estimate of largest eigenvalue $\lambda_{1}\to \frac{\left(\omega^{2}-1\right)}{2}>0$ ∀ *ω* > 1. Since the discriminant is negative ∀ *ε* > 0, the remaining complex conjugate eigenvalue pair have a negative real part given by $\text{Re}\left(\lambda_{2,3}\right)=\left(\text{tr}\left(J\right)-\lambda_{1}\right)/2=-(\frac{k}{2}+\frac{\left(\omega^{2}-1\right)}{4})$. Therefore, unlike in the previous example, we can see that origin here is unstable for large *ε*. The expression for ε* also shows that a higher value of *k* can extend the coupling range for a stable origin. This result is the first instance of unexpected behavior as we would normally expect a higher coupling value to keep the origin stable. Furthermore, in the *ε* > ε* regime it is numerically observed that the trajectories escape to infinity which is also quite unexpected.

One of the primary reasons behind considering augmented harmonic oscillator in this study is the fact that it is highly solvable and therefore can provide necessary insights into the physical mechanisms behind the desirable as well as undesirable behaviors. It turns out that in case of a successful stabilization, this system represents a forced harmonic oscillator where the steady state solution (which is completely determined by the forcing) decays to the origin along with an exponentially decaying force. Similarly, the case where the trajectories escape to infinity corresponds again to a forced system but this time being driven by an exponentially diverging force which is analogous to a situation where energy is being pumped into the system. Therefore the steady state solution in this case diverges along with the diverging force explaining the unexpected behavior of escaping trajectories observed for the fully augmented system. Details of the calculations leading to these deductions are available in Sec. Harmonic oscillator: diverging trajectories.

### Duffing oscillator

General equations for a linearly augmented Duffing oscillator with no damping or forcing can be written as:

$$\begin{array}{ccc} \dot{x} & = & y+\varepsilon_{1}u,\\ \dot{y} & = & x-x^{3}+\varepsilon_{2}u,\\ \dot{u} & = & -ku-\varepsilon_{1}\left(x-x^{*}\right)-\varepsilon_{2}\left(y-y^{*}\right). \end{array}$$ (8)

Uncoupled Duffing system has an invariant of motion (also the Hamiltonian) given by *H*(*x*, *y*) = *y* ^2^/2 − *x* ^2^/2 + *x* ^4^/4 and stationary solutions: (x*, y*) = (±1, 0), (0, 0). The trajectories of this system evolve on the double well potential surface of *H*(*x*, *y*) on starting with different initial conditions for *ε*_1_ = *ε*_2_ = 0. Similar to the previous example, for a successful stabilization, the required stationary solutions of the full system should be (x*, y*, u*) = (0, 0, 0) or (±1, 0, 0) where the system effectively decouples from the augmentation. These solutions will be referred to as (x*, y*) = (0, 0) (origin) or (±1, 0) in the following.

For the system in Eq 8, the characteristic eigenvalue equation can be expressed as,

$$\begin{array}{c} \left(\lambda +k\right)\left(\lambda^{2}+3{x^{*}}^{2}-1\right)+\lambda \left({\varepsilon_{1}}^{2}+{\varepsilon_{2}}^{2}\right)+\varepsilon_{1}\varepsilon_{2}\left(2-3{x^{*}}^{2}\right)=0. \end{array}$$ (9)

Now similar to the harmonic oscillator example, considering partial augmentation with *ε*_1(2)_ = *ε*, and *ε*_2(1)_ = 0 first, Eq 9 suggests that the stability characteristics for the stationary solutions are again independent of whether the system is being augmented in *x* or *y*. For this partial augmentation, Eq 9 gives,

$$\begin{array}{c} \left(\lambda +k\right)\left(\lambda^{2}+3{x^{*}}^{2}-1\right)+\lambda \varepsilon^{2}=0. \end{array}$$ (10)

**Fig 2:**
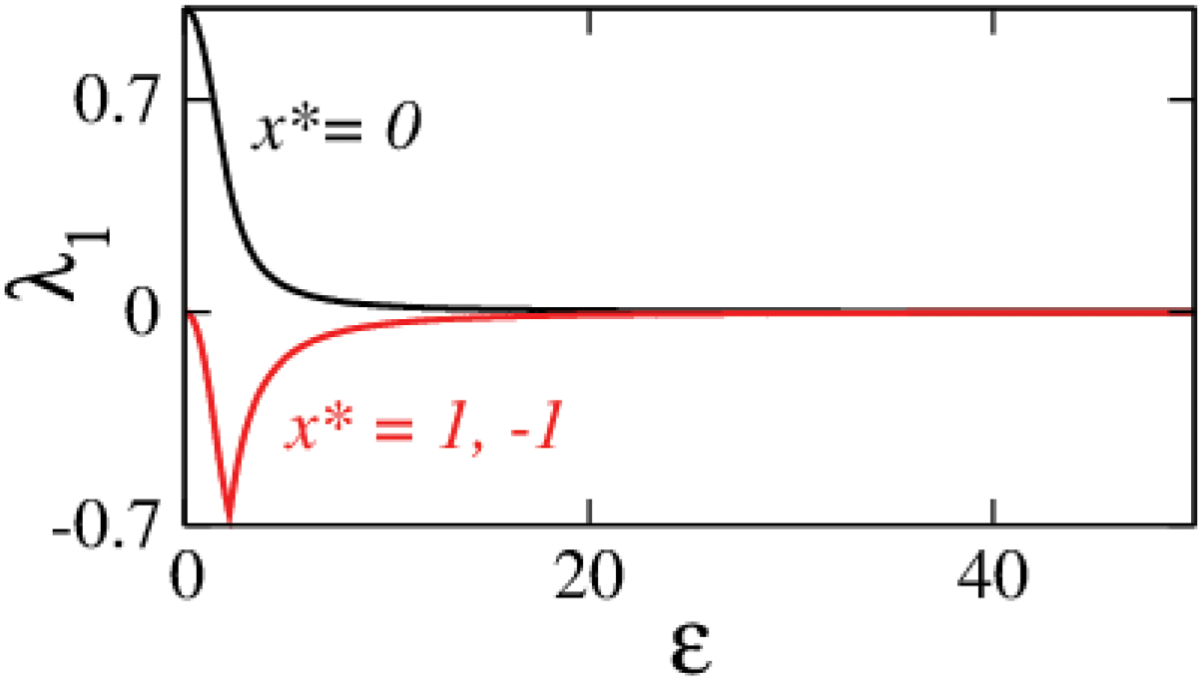
Behavior of the partially augmented Duffing oscillator. Largest eigenvalue estimates for stationary solutions (x*, y*) = (0, 0) (black), (x*, y*) = (±1, 0) (red).

Substituting x* = 0,±1, and rearranging the terms, we can obtain the characteristic eigenvalue equations for these stationary solutions as,

$$\begin{array}{c} \lambda^{3}+k\lambda^{2}+\left(\varepsilon^{2}-1\right)\lambda -k=0, \end{array}$$ (11)

for (x*, y*) = (0, 0) (hyperbolic for *ε* = 0), and

$$\begin{array}{c} \lambda^{3}+k\lambda^{2}+\left(\varepsilon^{2}+2\right)\lambda +2k=0, \end{array}$$ (12)

for (x*, y*) = (±1, 0) (non hyperbolic for *ε* = 0) respectively. It is straightforward to check that the largest eigenvalue *λ*_1_ → 0 for larger *ε* values in both these cases which implies that a stable/unstable stationary solution will stay the same until a stability change (zero crossing of the eigenvalue/s) occurs in the *ε* → ∞ limit. Furthermore using the RHC, it is easily verifiable that Eq 11 will always have positive root/roots, whereas Eq 12 will have all negative roots ∀ *ε* > 0; which implies that the x* = 0 is always unstable and x* = ±1 is always stable. Therefore, we see that partial augmentation works for stabilizing (x*, y*) = (±1, 0) but *fails completely* to stabilize the origin (x*, y*) = (0, 0). Fig 2 shows the largest eigenvalue calculations which verify these deductions. This brings us to an important observation that there might exist situations where it is not possible to target the desired stationary solution even on using an appropriate feedback function with any combination of *k* and *ε* values.

**Fig 3:**
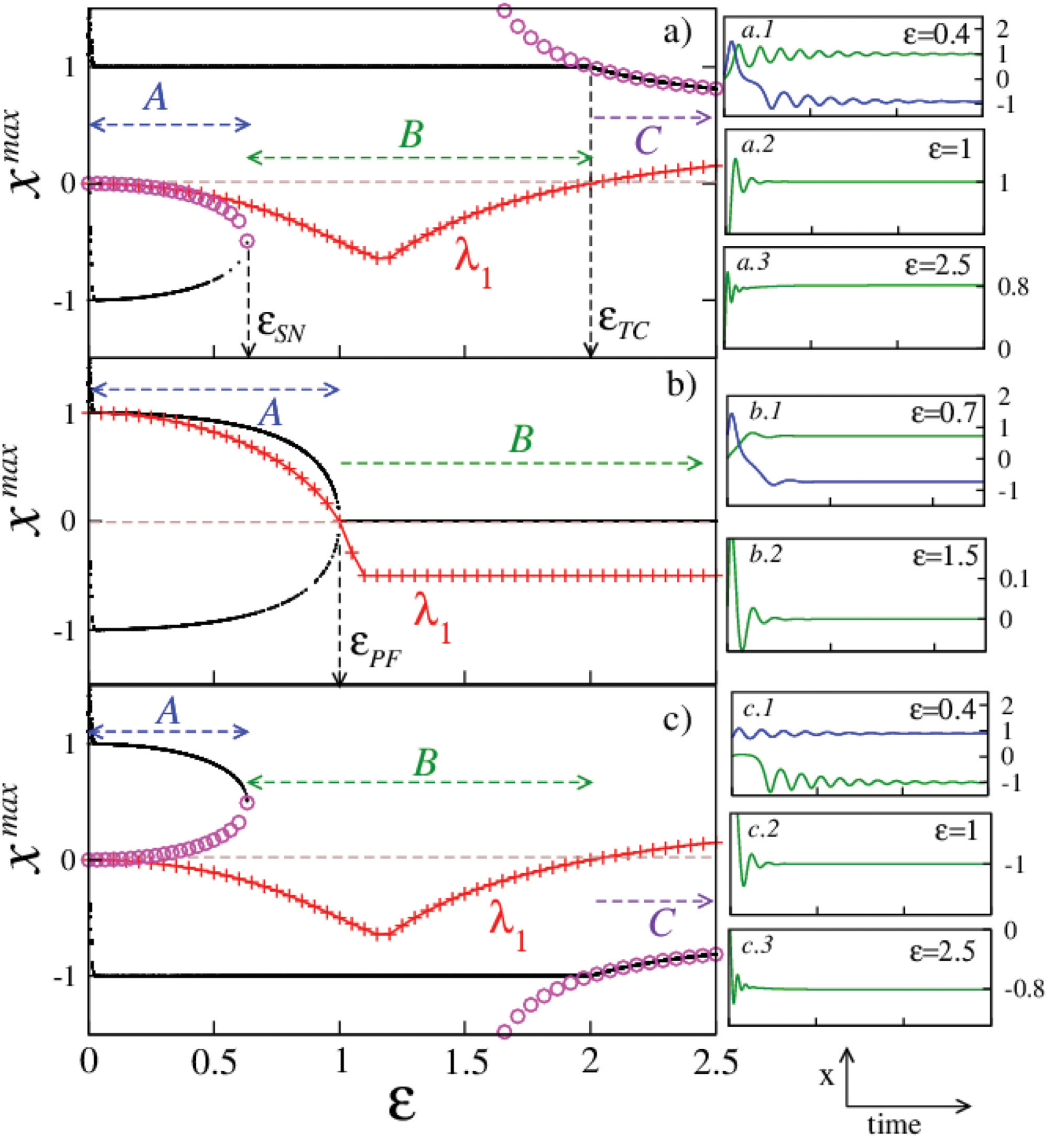
Different dynamical regimes in fully augmented Duffing oscillator. Figs a), b) and c) show the bifurcation diagrams (black dots) and the largest eigenvalues (red symbols) for (x*, y*) = (1, 0), (0, 0), and (−1, 0) respectively. Different dynamical regimes are marked as: *A* shows the regimes of bistability between different stationary solutions, *B* shows the regime where the desired stationary solution is the only dynamical attractor, and *C* marks the regime where other stationary solutions are stable. Circles mark the solution branchs (x*_+_, y*_+_, z*_+_) and (x*_−_, y*_−_, z*_−_) from Eq 13 in a) and c) respectively. Related Figs a.1: for *ε* = 0.4, the system is bistable and the two related transient behaviors (in blue and green and likewise for other cases), a.2: for *ε* = 1, the trajectory approaching the stable stationary solution (1, 0), and a.3 shows an arbitrary time series for *ε* = 2.5. Similarly in b.1: bistability, and in b.2: the system approaching the stable stationary solution (0, 0) is shown. Identically, c.1, c.2, and c.3 show bistability (*ε* = 0.4), stabilization of (−1, 0) (*ε* = 1) and an arbitrary time series at *ε* = 2.5 respectively.

Now considering identical augmentation with *ε*_1_ = *ε*_2_ = *ε* and we will see that this system has some interesting properties. Fig 3 shows the bifurcation diagrams of the system as we try targeting the different desired stationary solutions: For (x*, y*) = (1, 0), the bifurcation diagram (black dots) is shown in Fig 3a. Appropriate transient trajectories in different coupling regimes are also shown in related Fig 3(a.1), 3(a.2) and 3(a.3). It is observed that even for very small coupling values, the system quickly gets into a stable stationary state regime, although, for smaller values of *ε*, it exhibits bistability. The transient trajectories in this parameter regime are shown in Fig 3(a.1). We observe that the augmentation is stabilizing our desired stationary solution at (x*, y*) = (1, 0), but along with it, other stationary solutions which are *ε* dependent are also getting stabilized on starting with different initial conditions. These other stationary solutions for the augmented system here are given by,

$$\begin{array}{ccc} {x^{*}}_{\pm } & = & \frac{1}{2}\left(-1\pm \sqrt{1-\frac{4\varepsilon^{2}}{k-\varepsilon^{2}}}\right),\\ {y^{*}}_{\pm } & = & {x^{*}}_{\pm }-{{x^{*}}_{\pm }}^{3},\\ {u^{*}}_{\pm } & = & -\frac{{y^{*}}_{\pm }}{\varepsilon }, \end{array}$$ (13)

and solutions (x*_−_, y*_−_, z*_−_) are observed to coexist along with (x*, y*) = (1, 0). For higher coupling values, bistability terminates via a saddle node bifurcation when the stable branch of stationary solutions (x*_−_, y*_−_, u*_−_) collides with the unstable branch of (x*_+_, y*_+_, u*_+_)(circles) as shown in Fig 3a at $\varepsilon_{SN}=\sqrt{\frac{k}{5}}$. The system also exhibits hysteresis in this bistable regime and a brief discussion regarding this observation is available in Sec. Duffing system: hysteresis. Beyond this regime for a range of values in *ε* > *ε*_*SN*_, (x*, y*) = (1, 0) remains as the only stable attractor as shown in Fig 3(a) and 3(a.2).

In absence of augmentation, the eigenvalues for (x*, y*) = (1, 0) are complex: $\lambda_{1,2}=\pm i\sqrt{2}$. For the augmented system, the characteristic equation can therefore be written as:

$$\begin{array}{c} \lambda^{3}+k\lambda^{2}+2\lambda \left(1+\varepsilon^{2}\right)+\left(2k-\varepsilon^{2}\right)=0. \end{array}$$ (14)

The RHC shows that this equation will have all negative roots for (2*k* − *ε* ^2^)>0 and positive root/roots appear for $\varepsilon >\sqrt{2k}$. This gives us the transition threshold for the destabilization of the stationary solution as $\varepsilon^{*}=\sqrt{2k}$, at which the eigenvalue/s cross the zero axis. Since the discriminant is negative, the characteristic equation has one real and two complex conjugate roots. Considering large *ε* behavior, it is seen that the largest eigenvalue *λ*_1_ → 1/2 which implies that (x*, y*) = (1, 0) is unstable in this range. The real part of the remaining complex conjugate eigenvalue pair is Re(*λ*_2,3_) = −(2*k* + 1)/4. At ε*, Eq 14 can be rewritten as,

$$\begin{array}{c} \lambda (\lambda^{2}+k\lambda +2\left(1+2k\right))=0, \end{array}$$ (15)

which consequently gives the eigenvalues as *λ*_1_ = 0 and $\lambda_{2,3}=\left(-k\pm \sqrt{k^{2}-8\left(1+2k\right)}\right)/2$. We can see that *λ*_2,3_ will be a complex conjugate pair for $k\in (8-6\sqrt{2},8+6\sqrt{2})$. For our calculations, we have considered *k* = 2 which gives that at $\varepsilon^{*}=\sqrt{2k}=2$, *λ*_1_ crosses the zero line as can be seen in Fig 3a (red symbols). For higher values of $\varepsilon >\sqrt{2k}$, stationary states (x*_+_, y*_+_, u*_+_) (circles) in Fig 3(a) for *ε* > ε*(= *ε*_*TC*_) get stabilized via a transcritical bifurcation where (x*, y*) = (1, 0) and (x*_+_, y*_+_, u*_+_) exchange their stability. This again is quite *unexpected* since the controller is designed to stabilize (x*, y*) = (1, 0) for higher *ε* values. A brief discussion regarding the behavior of this system in the (*ε*, *k*) plane is available in Sec. Duffing system: (*ε*, *k*) plane behavior.

For the *origin* at (x*, y*) = (0, 0), the bifurcation diagram (black dots) is shown in Fig 3b. Appropriate transient trajectories corresponding to different augmentation regimes are also shown in related Fig 3(b.1) and 3(b.2). We observe bistability for a range of lower *ε* values before the origin gets stabilized. The stationary solutions obtained in the bistable regime are given by

$$\begin{array}{ccc} {x^{o}}_{\pm } & = & \pm \sqrt{1-\frac{\varepsilon^{2}}{k-\varepsilon^{2}}},\\ {y^{o}}_{\pm } & = & {x^{o}}_{\pm }-{{x^{o}}_{\pm }}^{3},\\ {u^{o}}_{\pm } & = & -\frac{{y^{o}}_{\pm }}{\varepsilon }. \end{array}$$ (16)

Transient trajectories in this regime demonstrating the two observed stationary solutions are shown in Fig 3(b.1). These solutions approach and collapse at the origin in a pitchfork bifurcation for $\varepsilon_{PF}=\sqrt{\frac{k}{2}}$ (= 1 for *k* = 2 in this case) beyond which the solutions (*x* ^*o*^_±_, *y* ^*o*^_±_, *u* ^*o*^_±_) become imaginary and the origin is the only stable real stationary solution. A transient trajectory in this parameter regime is shown in Fig 3(b.2).

The characteristic equation for the origin is,

$$\begin{array}{c} \lambda^{3}+k\lambda^{2}+\lambda \left(2\varepsilon^{2}-1\right)+2\varepsilon^{2}-k=0, \end{array}$$ (17)

which has all negative eigenvalues for 2*ε* ^2^−*k* > 0 giving us a stability regime of $\varepsilon >\sqrt{k/2}$ and a transition value of $\varepsilon_{PF}=\varepsilon^{*}=\sqrt{k/2}=1$ (since *k* = 2) when the eigenvalue/s cross the zero axis. From Eq 17, we get *λ*_1_ = −1 in the large *ε* limit. It is numerically observed here that the discriminant Δ < 0 in this range and therefore Re(*λ*_2,3_) = (1−*k*)/2 = −0.5 and consequently, the origin is stable in the large *ε* limit. The largest eigenvalue for the origin is plotted as red symbols in Fig 3(b) which shows the changes in the stability of the origin from unstable in *ε* ∈ (0, 1) to stable ∀ *ε* > 1. For a discussion regarding the system behavior in the (*ε*, *k*) plane, please again see Sec. Duffing system: (*ε*, *k*) plane behavior for details.

For (x*, y*) = (−1, 0), the bifurcation diagram (black dots) is shown in Fig 3c. Appropriate transient trajectories corresponding to different augmentation regimes are also shown in related Fig 3(c.1), 3(c.2) and (c.3). Since this solution is a symmetric counterpart of (x*, y*) = (1, 0), the corresponding analysis similarly carries over in this case.

These simple examples demonstrate the fact that targeting the required stationary solutions using linear augmentation is not quite straightforward and the procedure is quite sensitive to how the systems are augmented, the stationary solutions being targeted and to the properties of systems. In the following, results for a specific class of dissipative dynamical systems are presented to further highlight these limitations.

### Dissipative predator—prey models

Considering predator—prey population models, general evolution equations for these systems with logistic prey growth can be written as,

$$\begin{array}{ccc} \dot{x} & = & rx(1-x/K)-f(x)y,\\ \dot{y} & = & (\rho f(x)-\gamma )y, \end{array}$$ (18)

where *x* and *y* correspond to prey and predator populations respectively and the parameters *r*, *K*, *ρ*, and *γ* are positive. Considering the evolution equation for preys, the first term *rx*(1 − *x*/*K*) represents the logistic growth rate of the prey species with the maximum growth rate of *r* and carrying capacity *K* which is the maximum population size that the environment can sustain indefinitely. The second term *f*(*x*)*y* corresponds to the prey mortality via predation. *f*(*x*) is the functional response governing the rate of per capita prey consumption by the predators [39–41]. The parameter *ρ* governs the biomass conversion efficiency for the predators in the sense of how many predators are added to the population via predation, and *γ* is the intrinsic predator mortality parameter. One of the stationary solutions of this system corresponds to vanishing predator-prey populations, i.e. (x*, y*) = (0, 0). The other stationary solutions are dependent on the type of functional response considered. Most commonly employed *f*(*x*) forms in such models are the Holling type with the following general expressions:

1. *f*(*x*) = *ax* for Holling type I response which is identical to the predation in the Lotka—Volterra case [42, 43],
2. $f\left(x\right)=\frac{ax}{\left(b+x\right)}$ for Holling type II (Michaelis—Menten kinetics), using which, Eq 18 gives the Rosenzweig—MacArthur model [44],
3. $f\left(x\right)=\frac{ax^{2}}{\left(b^{2}+x^{2}\right)}$ for Holling type III (Hill equation type), using which, Eq 18 gives the Truscott—Brindley model [45] which is used in modeling phytoplankton and zooplankton interactions leading to harmful algal blooms,

and consequently, corresponding stationary solutions can be obtained. The parameter *a* in expressions above corresponds to the maximum per capita predation rate, and *b* is the half saturation constant governing how quickly the predators attain their maximum consumption rate. In the following, we will have a closer look at the stability properties of the trivial stationary solution (x*, y*) = (0, 0): *origin*. Considering a general augmented population model,

$$\begin{array}{ccc} \dot{x} & = & rx\left(1-x/K\right)-f\left(x\right)y+\varepsilon_{1}u,\\ \dot{y} & = & \left(\rho f\left(x\right)-\gamma \right)y+\varepsilon_{2}u,\\ \dot{u} & = & -ku-\varepsilon_{1}\left(x-x^{*}\right)-\varepsilon_{2}\left(y-y^{*}\right), \end{array}$$ (19)

it turns out that the Jacobian for this system is identical for all three functional responses at the origin. The identical characteristic equation therefore is,

$$\begin{array}{c} \left(r-\lambda \right)\left(\gamma +\lambda \right)\left(k+\lambda \right)+{\varepsilon_{2}}^{2}\left(r-\lambda \right)-{\varepsilon_{1}}^{2}\left(\gamma +\lambda \right)=0. \end{array}$$ (20)

For *ε*_1_ = *ε*_2_ = 0, we obtain the eigenvalues as *λ*_1_ = *r*, *λ*_2_ = −*γ*, and *λ*_3_ = −*k* where *λ*_1_ and *λ*_2_ are the eigenvalues for the original system in Eq 18 implying that the origin is unstable, and *λ*_3_ corresponds to the exponentially decaying control variable *u*. Since the Holling type I case with insatiable predators is quite unrealistic, we will focus here on systems with Holling type II (H II) and III (H III) behaviors. In the following analysis, the parameter values are fixed at: *r* = 0.5, *K* = 0.5, *a* = 1/3, *b* = 1/15, *ρ* = 0.5, *γ* = 0.1 for the H II [46] system, and *r* = 0.43, *K* = 1, *a* = 1, *b* = 0.053, *ρ* = 0.05, *γ* = 0.028 for H III [45, 47]. Let us now look at different augmentation situations.

For *ε*_1_ = *ε* and *ε*_2_ = 0, i.e. only prey populations are augmented, substituting these values in Eq 20 gives,

$$\begin{array}{c} \left(\gamma +\lambda \right)[\left(r-\lambda \right)\left(k+\lambda \right)-\varepsilon^{2}]=0. \end{array}$$ (21)

**Fig 4:**
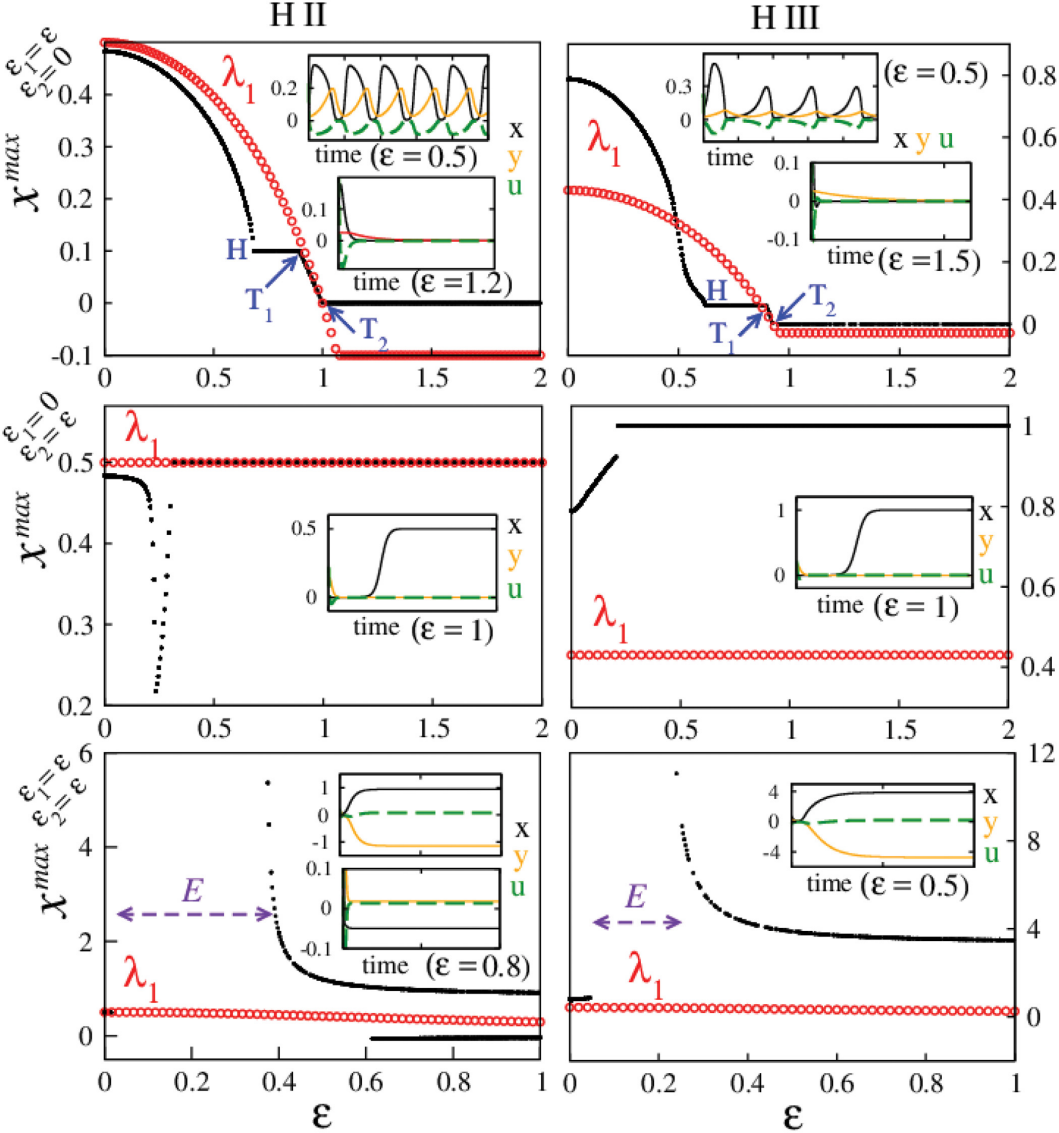
Augmented predator—prey model dynamics. Bifurcation diagram (black dots), largest eigenvalues (red circles) and time series (insets for specific *ε* values) for predator—prey systems with H II (left column) and H III (right column) functional responses. Top row: For augmented prey, insets show the time series of *x*, *y* and *u* for two different *ε* values before (with oscillatory *u*) and after the stabilization of (x*, y*) = (0, 0) (with u* = 0). The Hopf bifurcation is marked as *H*, and the transcritical bifurcation points have been highlighted by *T*_1_, and *T*_2_ respectively. Middle row: For augmented predator, the systems exhibit oscillatory behavior similar to augmented preys (not shown) for low *ε* before they settle on the stationary solution (x*, y*) = (*K*, 0) with u* = 0; where the preys reach their carrying capacity in the absence of predators for higher *ε* values. Bottom row: For augmented predator and prey, for low *ε*, the systems are oscillatory (not shown). With increasing *ε*, both the systems lose the oscillatory behavior and all trajectories escape to infinity in the regime marked by *E*. For higher *ε* values, unrealistic stationary solutions where either the preys exceed their carrying capacity (x* > *K*) with negative predator populations (y* < 0) (H II and H III), or where the prey populations are negative with small positive predator population (for H II) and u* ≠ 0 get stabilized.

Since one of the roots *λ* = −*γ* is independent of *ε* therefore the remaining roots of this equation determine the stability of the origin. The remaining two roots are $\lambda_{\pm }=-\left(k-r\right)/2\pm \sqrt{{\left(k+r\right)}^{2}-4\varepsilon^{2}}/2$ out of which, *λ*_−_ < 0, ∀ *ε*. It is easily verifiable that the eigenvalue *λ*_+_ (which also is the largest) is positive ∀ $\varepsilon <\sqrt{kr}$ and crosses the zero axis at $\varepsilon^{*}=\sqrt{kr}$ leading to all negative eigenvalues and hence a stable origin. This is quite similar to the harmonic oscillator case where increasing/decreasing the value of the decay parameter *k* could increase/decrease the threshold value of stable → unstable transition (unstable → stable in this case). Furthermore, in the large *ε* limit, we obtain the largest eigenvalue *λ*_1_ = −*γ* and therefore the origin is stable in this regime. Fig 4: top row shows the bifurcation diagram and the largest eigenvalue behavior for H II (left) and III (right). The unstable → stable transition in both these systems with increasing coupling values can be seen in the figure. Although for the H III system in the regime $\varepsilon >\sqrt{rk}(=0.927,$ for *r* = 0.43, *k* = 2), certain initial conditions lead to the trajectories escaping to infinity (not shown) which accounts for the missing dots in the bifurcation figure. Since *x* and *y* are population variables by definition, population models are constrained to work for non-negative values of *x* and *y* respectively. What we observe here is that the augmentation forces the prey populations into negative values which leads to a breakdown in the model constraints and the logistic function in the rate equation of *x* leads to diverging solutions as time increases. For H II system this appears not to be the case and all considered initial conditions lead to a stable origin ∀ $\varepsilon >\sqrt{rk}(=1,$ for *r* = 0.5, *k* = 2).

For *ε*_1_ = 0 and *ε*_2_ = *ε*, i.e. only the predator populations are augmented, substituting these values in Eq 20 gives,

$$\begin{array}{c} \left(r-\lambda \right)[\left(\gamma +\lambda \right)\left(k+\lambda \right)+\varepsilon^{2}]=0, \end{array}$$ (22)

and we see that an eigenvalue *λ* = *r* is always positive since *r* > 0, and therefore this setup will *never* stabilize the origin. The remaining eigenvalues are $\lambda_{\pm }=-\left(\gamma +k\right)/2\pm \sqrt{\left(k-\gamma \right)+4\varepsilon^{2}}/2$. In Fig 4: second row, for low *ε* values, the systems exhibit periodic behavior similar to the one shown for the augmented prey case. For higher values of *ε*, both H II and H III settle on a stationary solution of the original system (x*, y*) = (*K*, 0) which we did not intent to stabilize. For this solution, the preys exist at their carrying capacity and the predators vanish. For H II and H III, the carrying capacities considered for simulations are *K* = 0.5 and *K* = 1 respectively, and hence the observations in Fig 4 (middle row).

For *ε*_1_ = *ε*_2_ = *ε*, i.e. both prey and predator populations are augmented, substituting these values in Eq 20 and rearranging terms gives,

$$\begin{array}{c} \lambda^{3}+\left(\gamma +k-r\right)\lambda^{2}+\left(2\varepsilon^{2}+\left(\gamma -r\right)k-r\gamma \right)\lambda +\varepsilon^{2}\left(\gamma -r\right)-r\gamma k=0. \end{array}$$

Using the RHC, one of the conditions for this equation to have all negative roots is $\varepsilon >\sqrt{\frac{r\gamma k}{\left(\gamma -r\right)}}$ which is impossible to achieve since *r* > *γ*. Therefore, this setup will not stabilize the origin either. Fig 4: bottom row shows the behavior of H II and H III. For smaller *ε* values, these systems exhibit periodic behavior similar to the augmented prey. On increasing *ε* further, systems enter a regime where all considered initial conditions lead to escaping trajectories. The reason behind this behavior here again is due to a breakdown in modeling constraints. Examination of transient trajectories reveals that augmentation in this case is forcing the predator populations into *y* < 0 axis which leads to a breakdown in the model, thereby initiating a positive feedback loop in the prey populations leading to the diverging behaviors observed in simulations. Beyond this regime for higher values of *ε*, H II system exhibits bistability between different stationary solutions where in one case, preys exceed their carrying capacity (x* > *K*) and the predator populations are negative (y* < 0), and in the other case, the prey populations are negative (x* < 0) and predators assume a small positive value. It is important to note yet again that these solutions are impractical because the populations cannot exist above their carrying capacities nor can they take negative values. For H III system, we only observe the equilibrium solutions with x* > *K* and y* < 0 (see inset). In both the cases, we have a non vanishing u* > 0 and therefore these solutions exist due to augmentation and cannot be observed otherwise. Following this analysis, we can conclude that augmenting the prey is the correct strategy to stabilize of the origin and the other coupling schemes can lead to complicated dynamics. Even though the analysis here is limited to the origin, we can expect these behaviors to be quite general with respect to other stationary solutions as well.

**Fig 5:**
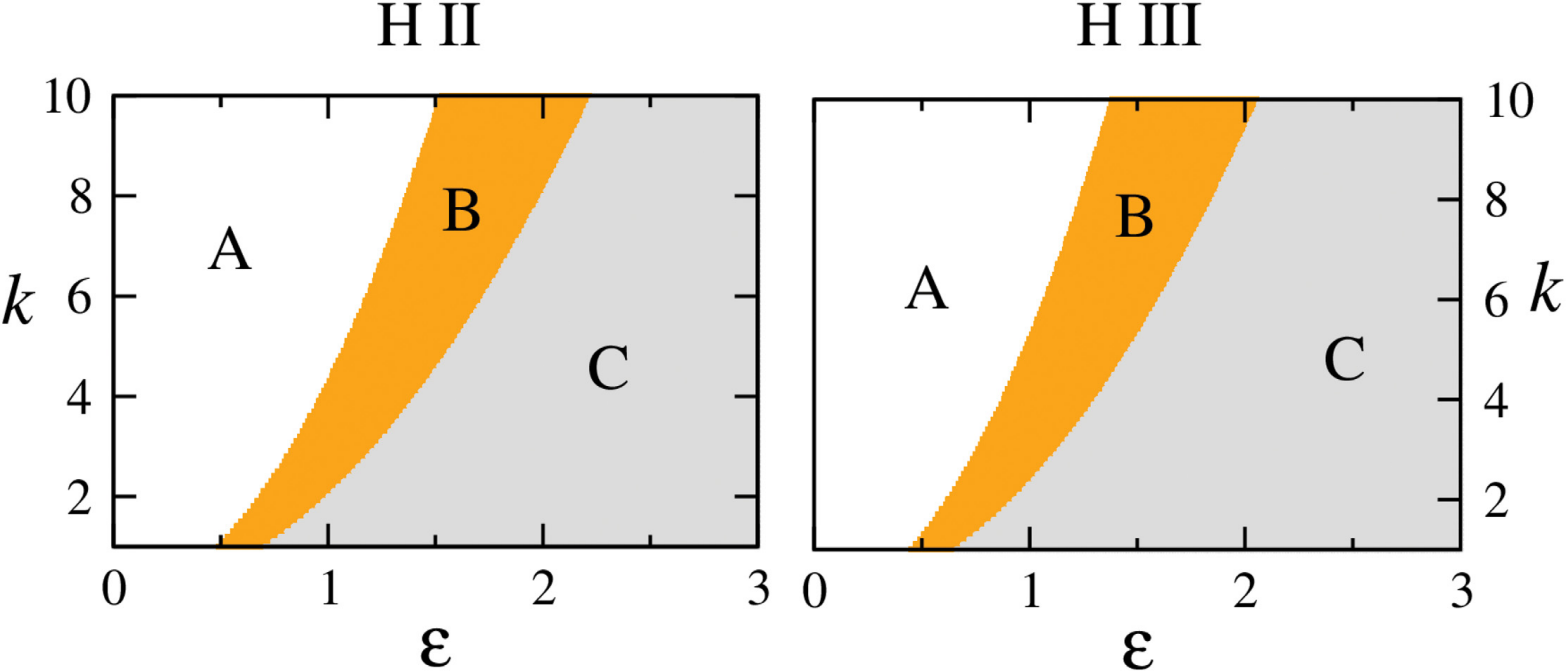
Different dynamical regimes for H II and H III systems. Regimes are marked as A, B and C in the *ε*, *k* plane. A is the regime of periodic dynamics, in B stationary solutions of the coupled system are stable and C is the regime of stable origin. The boundaries between A→B and B→C are the loci of the reverse Hopf bifurcation *H* and the second transcritical bifurcation *T*_2_ respectively (as in Fig 4: top row).

Now considering applications, as already mentioned, origin corresponds to an equilibrium for which the predators and the preys vanish. Persistence of populations for a proper ecosystem function is very imperative and has been studied extensively from several perspectives, contributing towards a better understanding of the processes leading to species extinction [48–52]. Knowledge regarding these processes can help in devising procedures which can contribute towards better species conservation efforts. For the simple models considered in the previous analysis, it is clear that either by coupling the system appropriately or by using specific parameter values for *k* and *ε*, we can avoid stabilizing the origin. For instance, considering the prey augmented case, for low *ε*, the systems exhibit periodic oscillations. On increasing the coupling strength, stationary solutions of the augmented system (x* > 0, y* > 0, u* < 0), satisfying

$$\begin{array}{ccc} rx^{*}\left(1-x^{*}/K\right)-f\left(x^{*}\right)y^{*}+\varepsilon_{1}u^{*} & = & 0,\\ \left(\rho f\left(x^{*}\right)-\gamma \right)y^{*} & = & 0,\\ -ku^{*}-\varepsilon x^{*} & = & 0, \end{array}$$ (23)

get stabilized through a reverse Hopf bifurcation (marked as *H* in Fig 4 (top row)). For these stationary solutions, the value of x* stays constant while y* and u* = −εx*/*k* show a variation for a range of *ε* values (plateau between *H* and *T*_1_ in Fig 4 (top row)). It is also important to note that some initial conditions in this regime can lead to trajectories escaping to infinity. This branch of solutions undergoes a transcritical bifurcation (marked *T*_1_ in Fig 4 (top row)) where it exchanges stability with another branch of solutions with u* → 0 for increasing *ε*. At $\varepsilon^{*}=\sqrt{rk}$, u* = 0 and the predator—prey system effectively decouples from augmentation which is accompanied by another transcritical bifurcation (*T*_2_ in Fig 4 (top row)) between the continuing branch of stationary solutions (x* > 0, y* > 0, u* → 0) and the origin. In *ε* > ε* regime, origin is the only dynamical attractor. Fig 5 shows the parameter scans for H II and H III systems highlighting these different dynamical regimes. In region A these systems exhibit periodic behavior and the boundary between A and B is the locus of the Hopf bifurcation in the *ε*, *k* plane, which leads to the stabilization of stationary solutions (x* > 0, y* > 0, u* < 0). B corresponds to the regime where stable stationary solutions (x* > 0, y* > 0, u* < 0) and (x* > 0, y* > 0, u* → 0) are observed and the boundary between B and C is the locus of the second transcritical bifurcation *T*_2_ which leads to the stabilization of the origin. Therefore by using appropriate values of *ε* and *k*, we can keep the system in either a periodic state, or a stationary state with non vanishing populations and can expect this control to work in experiments and be robust with respect to demographic noise; Ref. [26] experimentally stabilized a stationary solution in an electronic Lorenz system at permitted noise level. Furthermore, in the other instances of augmented predators, or augmented predators and preys we already observe a complete lack of origin stabilization. Therefore, we can employ these schemes as well to avoid stabilizing the origin but one needs to be careful since these cases can lead to other complications as discussed. Another useful application for these observations could be in cases where maximization of prey yield is required. Augmenting the predator populations is seen to stabilize the equilibrium where the prey populations exist at their carrying capacity and the predators vanish. This can find applications in fisheries [53, 54], algae fuel generation [55, 56]; where maximal sustainable yields are crucial, and also in biomedical research, for e.g. in HIV-1 infection models [57] where a portion of human immune system i.e. activated CD4^+^ T cells are the primary target of the HIV-1 infection [58, 59] which can be modeled via predator—prey dynamics.

### Additional details

This section presents certain dynamical aspects related to augmented harmonic and Duffing oscillators for completeness. These have been compiled in a separate section to preserve the manuscript flow and for those who might be interested in these details.

#### Harmonic oscillator: diverging trajectories

Augmented harmonic oscillator dynamics from Eq 5 can also be expressed in form of a second order ODE as,

$$\begin{array}{c} Dx=U\left(\varepsilon_{1},\varepsilon_{2},k,t\right)\left[=\left(\varepsilon_{2}+\varepsilon_{2}{\varepsilon_{1}}^{2}-\varepsilon_{1}k\right)u\left(t\right)\right], \end{array}$$ (24)

where the derivative operator $D=D^{2}+\varepsilon_{1}\varepsilon_{2}D+\left(\varepsilon_{1}^{2}+\omega^{2}\right)$ with $D^{i}=\frac{d^{i}}{dt^{i}}$, *i* = 1, 2 in this case. This equation corresponds to a driven harmonic oscillator with frequency $\left(\varepsilon_{1}^{2}+\omega^{2}\right)$ and a damping coefficient *ε*_1_ *ε*_2_. The roots of the auxiliary equation for the operator D are *m*_±_ = *α* ± *β* where *α* = −*ε*_1_ *ε*_2_/2 and $\beta =\sqrt{\varepsilon_{1}^{2}\varepsilon_{2}^{2}-4\left(\varepsilon_{1}^{2}+\omega^{2}\right)}/2$. For partially augmented cases *α* = 0 and $\beta =\sqrt{-4(\varepsilon_{1}^{2}+\omega^{2})}/2$ or *β* = *iω* for *ε*_2_ = 0, *ε*_1_ ≠ 0 and *ε*_1_ = 0, *ε*_2_ ≠ 0 respectively.

For identical augmentation *ε*_1_ = *ε*_2_ = *ε*, we get $D=D^{2}+\varepsilon^{2}D+\left(\varepsilon^{2}+\omega^{2}\right)$ and Eq 24 reads

$$\begin{array}{c} Dx=U\left(\varepsilon ,k,t\right)[=\varepsilon \left(1+\varepsilon^{2}-k\right)u\left(t\right)]. \end{array}$$ (25)

**Fig 6:**
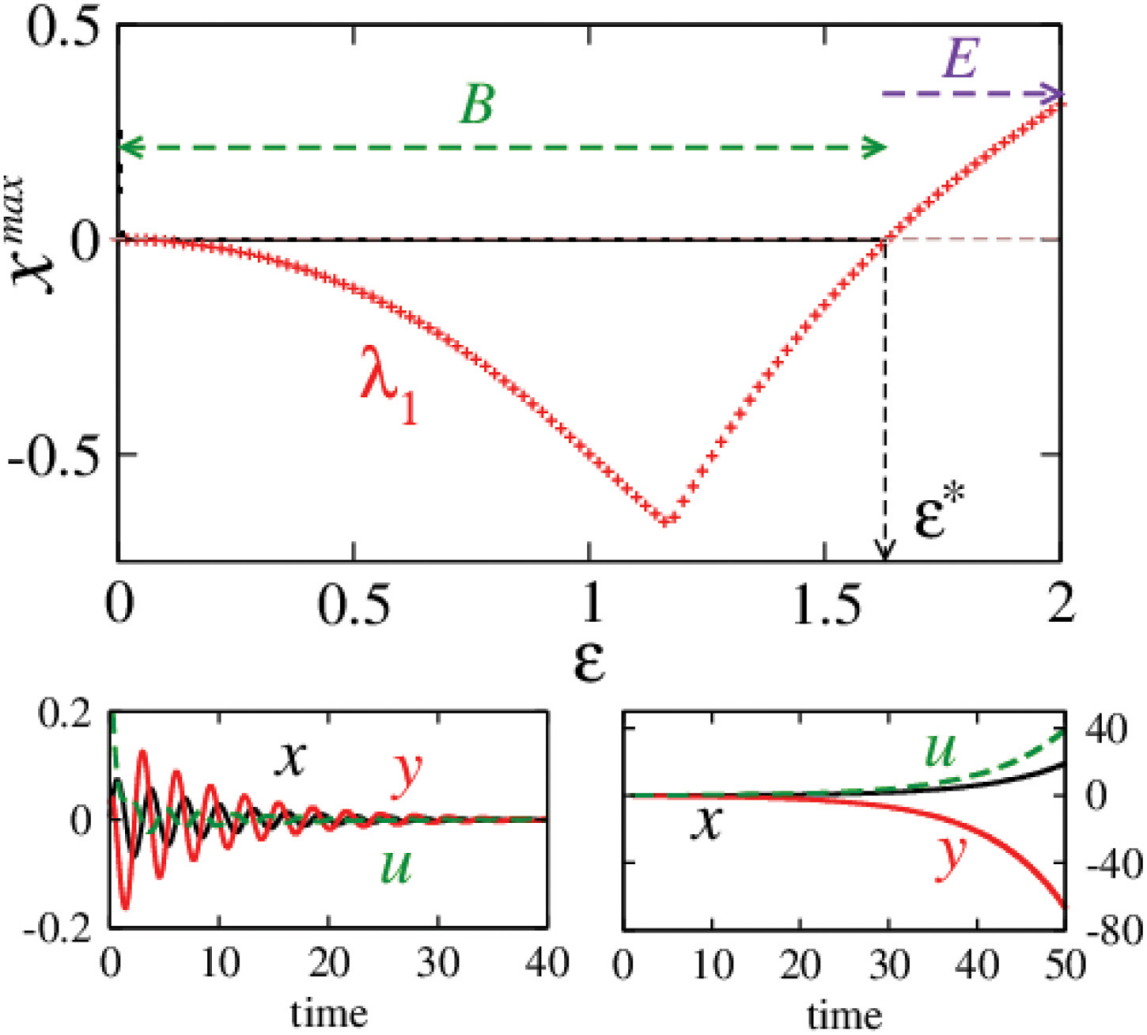
Transition between regimes of stable origin and escaping trajectories. Bifurcation diagram (black dots) along with the largest eigenvalue (red symbols) as a function of *ε* in the top row. $\varepsilon^{*}=\omega \sqrt{\frac{k}{\omega^{2}-1}}\left(∼1.63\right)$ marks the coupling beyond which all initial conditions lead to escaping trajectories in region *E*. Transient trajectories shown for *ε*(= 0.5) < ε* (left bottom) and *ε*(= 1.75) > ε* (right bottom).

The roots of the auxilliary equation in this case are *m*_±_ = *α* ± *β* with *α* = −*ε* ^2^/2 and $\beta =\sqrt{\varepsilon^{4}-4\left(\varepsilon^{2}+\omega^{2}\right)}/2$. For imaginary *β*, the transient solution for Eq 25 can be expressed as,

$$\begin{array}{c} x_{t}\left(t\right)=Ax_{1}\left(t\right)+Bx_{2}\left(t\right), \end{array}$$ (26)

which is independent of the forcing term *U*(*ε*, *k*, *t*) with *x*_1_(*t*) = exp(*αt*)cos*βt*, and *x*_2_(*t*) = exp(*αt*)sin*βt*. Consequently, the steady state solution can be obtained by using the Laplace and inverse Laplace transformations giving,

$$\begin{array}{c} x_{st}\left(t\right)=\frac{1}{\Omega }\int_{0}^{t}e^{-\gamma (t-x)}\sin\left(\Omega (t-x)\right)U\left(\varepsilon ,k,x\right)dx, \end{array}$$ (27)

where $\Omega =\sqrt{\omega_{0}^{2}-\gamma^{2}}$, ${\omega_{0}}^{2}=\varepsilon^{2}+\omega^{2}$ and *γ* = *ε* ^2^/2. Now at this point, we do not know the exact expression for *U*(*ε*, *k*, *t*). Considering the transient behavior of trajectories in partially/fully augmented system, we clearly observe that they possess an exponentially decaying/diverging envelop (see Fig 1 (inset) and Fig 6 bottom row). Based on these observations, assuming *U*(*ε*, *k*, *t*) = *a*_0_ exp (*k*_*m*_ *t*) where both *a*_0_, *k*_*m*_ are functions of *ε* and *k*, and solving Eq 27 gives the particular solution

$$\begin{array}{c} x_{p}\left(t\right)=\exp\left(k_{m}t\right)\left(\frac{a_{0}}{{\left(k_{m}+\alpha \right)}^{2}-\beta^{2}}\right). \end{array}$$ (28)

From this expression we see that the trajectories will exponentially decay to the origin ∀ *k*_*m*_ < 0 and diverge ∀ *k*_*m*_ > 0.

**Fig 7:**
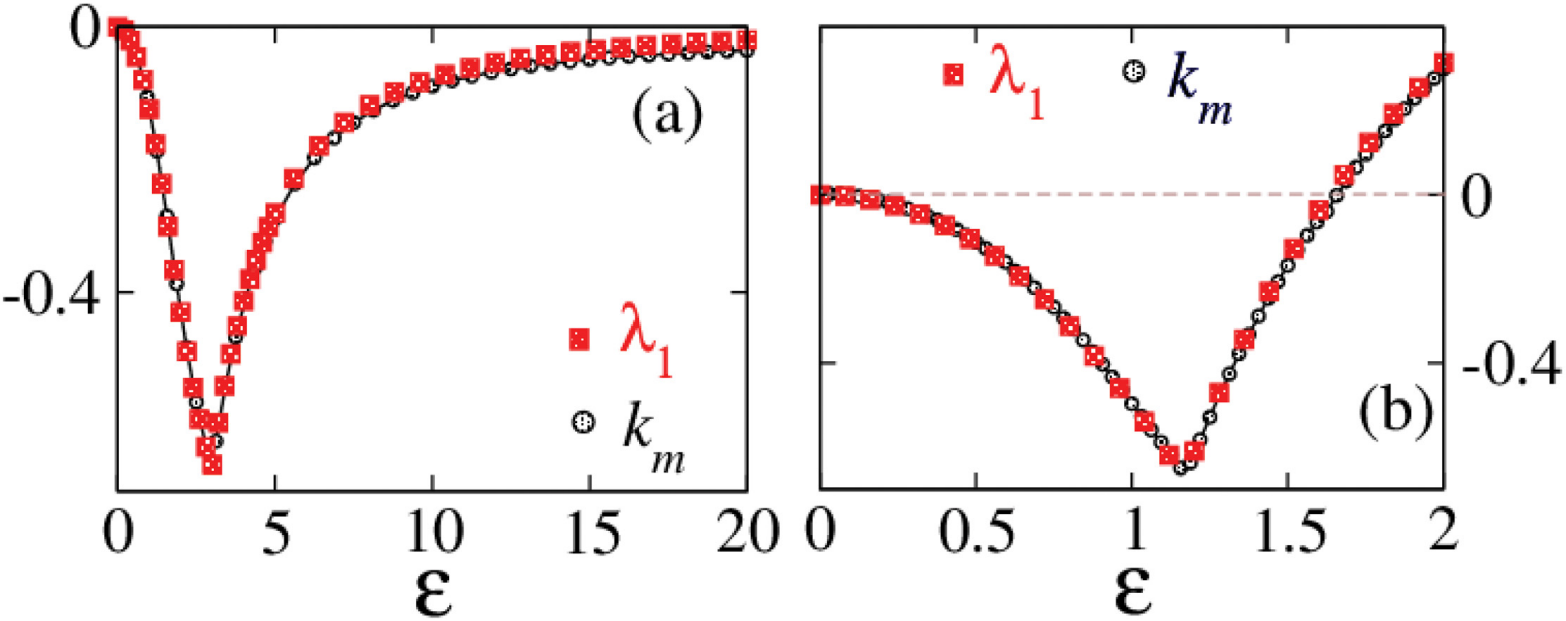
Largest eigenvalues and estimated values of *k*_*m*_ as functions of *ε*. (a) for the system augmented in *x* and (b) for the fully augmented system.

Fig 7 shows the numerical estimation of *k*_*m*_ along with the largest eigenvalue of the Jacobian *λ*_1_ at the origin as a function of *ε*; for partially (Fig 7(a)) and fully augmented cases (Fig 7(b)). *k*_*m*_ here was calculated as the average rate of convergence/divergence in the Euclidean distance of the current systems’ state from its previous state, for every time step along the trajectory. These results suggest that *k*_*m*_ = *λ*_1_ and this observation has some interesting consequences. For *k*_*m*_ = *λ*_1_ < 0, we have a case of a harmonic oscillator being driven by an exponentially decaying force and consequently the system settles on the origin as *t* → ∞. Similarly the other case of an unstable origin (*k*_*m*_ = *λ*_1_ > 0) is equivalent to the oscillator under the influence of an exponentially diverging force (energy being pumped into the system) which leads to diverging trajectories as time increases.

#### Duffing system: hysteresis

**Fig 8:**
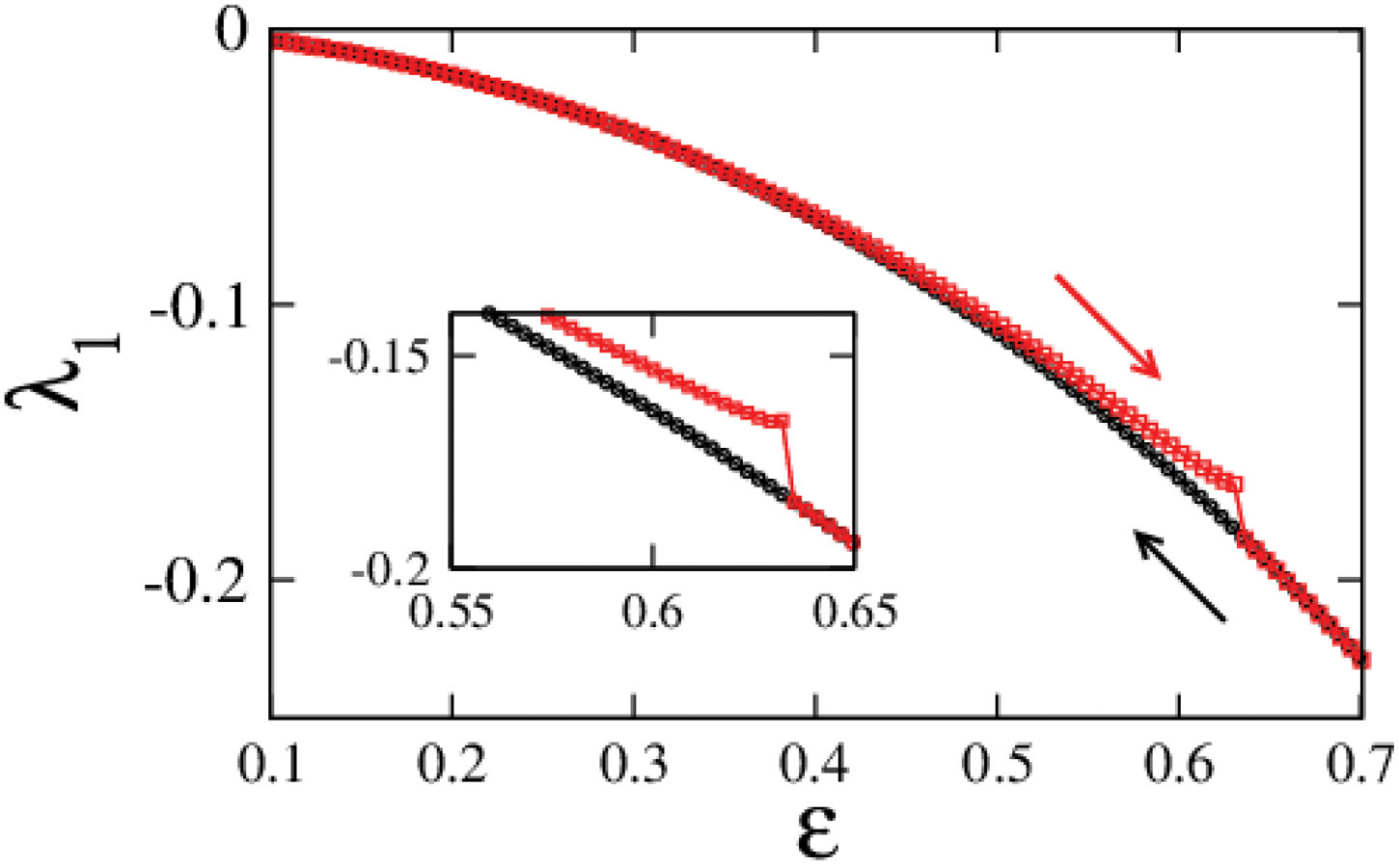
Hysteresis in the bistable regime. Largest Lyapunov exponent for increasing and decreasing values of *ε* for the fully augmented Duffing system with (x* = 1, y* = 0) demonstrating hysteresis. Calculations for increasing and decreasing *ε* are marked by red and black arrows respectively.

For the bistable, completely and identically augmented Duffing system in Eq 8 with x* = 1, y* = 0, the largest Lyapunov exponent was calculated for increasing and decreasing values of augmentation strength *ε*. Starting with initial conditions leading to the solutions (x*_−_, y*_−_, z*_−_) at *ε* = 0.1, the initial conditions for the next calculation at *ε* = 0.1 + *δε* were considered as the final values of *x*, *y*, *u* from the previous calculation for *ε* = 0.1, with *δε* = 0.001 and so on for the entire range in the forward direction. Similarly for backwards calculation, the process was repeated starting from *ε* = 0.7 where (1, 0) is the only stable solution with *δε* = −0.001. The results of the calculation are shown in Fig 8 and as one would expect, this system exhibits hysteresis in the interval of bistablity.

#### Duffing system: (*ε*, *k*) plane behavior

**Fig 9:**
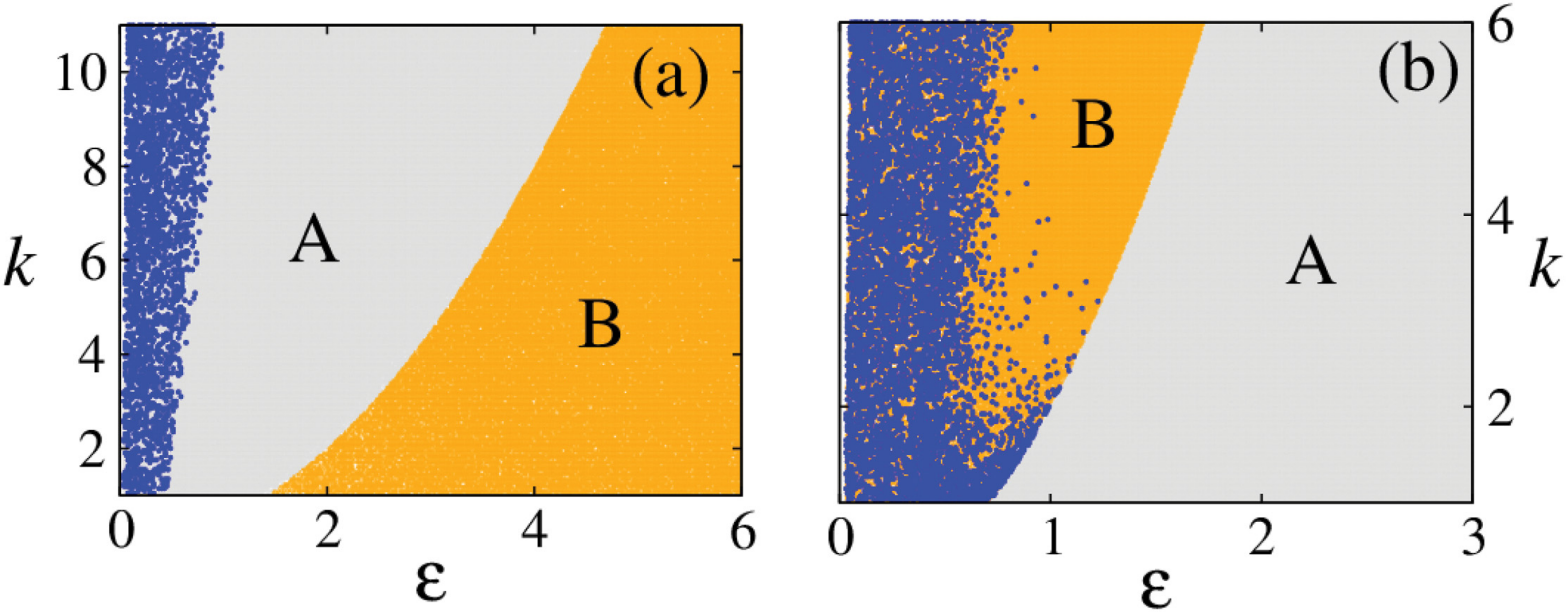
Behavior of fully augmented Duffing system in the (*ε*, *k*) plane. Parameter regimes marked in grey (A) correspond to a successful stabilization of the intended stationary solution; (1, 0) in (a) and (0, 0) in (b). Regimes of bistability are marked with blue dots and B corresponds to parameter values for which other stationary solutions are stable.

Different dynamical regimes for the Duffing system in Eq 8 with (x*, y*) = (1, 0) and (0, 0) are shown in Fig 9. The grey areas marked as A correspond to the regimes where the intended stationary solutions are successfully stabilized, namely (1, 0) and (0, 0) in Fig 9(a) and 9(b) respectively.

For x* = 1, y* = 0 in Fig 9(a): blue dots for lower *ε* values highlight the regimes of bistability where solutions (x*_−_, y*_−_, z*_−_) (from Eq 13) and (1, 0) coexist. Solutions (x*_−_, y*_−_, z*_−_) vanish via a saddle node bifurcation after colliding with the unstable branch of solutions (x*_+_, y*_+_, z*_+_) (again from Eq 13) and the boundary of the blue dot regime gives the locus of this saddle node bifurcation; parabolic function *F*_*SN*_(*ε*, *k*)(= 5*ε* ^2^−*k*) = 0, estimated from the expression for x*_±_ as the limiting value of *k* and *ε* to get real solutions, which is obtained by equating the discriminant in the expression of x*_±_ to zero. The boundary between regimes A and B corresponds to the locus of the transcritical bifurcation between (1, 0) and (x*_+_, y*_+_, z*_+_), and is given by the zero crossing of the largest eigenvalue for (1, 0) which satisfies *F*_*TC*_(*ε*, *k*)(= 2*ε* ^2^−*k*) = 0. In region B either the now stable coupling dependent stationary solutions (x*_+_, y*_+_, z*_+_), or escaping trajectories are observed.

Similarly for x* = 0, y* = 0 in Fig 9(b): B highlights the regime where the system settles on the solutions (*x* ^*o*^_+_, *y* ^*o*^_+_, *u* ^*o*^_+_) from Eq 16. The blue dots correspond to the initial conditions leading to stationary solutions (*x* ^*o*^_−_, *y* ^*o*^_−_, *u* ^*o*^_−_) also from Eq 16. Since the system is bistable in this regime, we should expect the entire region B to be filled with these blue dots but that is not the case. The reason behind this behavior is a difference in the relative basin size of these two solutions; number of initial conditions leading to (*x* ^*o*^_+_, *y* ^*o*^_+_, *u* ^*o*^_+_) is more than the ones which lead to (*x* ^*o*^_−_, *y* ^*o*^_−_, *u* ^*o*^_−_). This difference is even more pronounced for higher values of *k*. Both these solutions vanish via a pitchfork bifurcation and the locus of this bifurcation which separates regimes B and A is defined by the function *F*_*PF*_(*ε*, *k*)(= 2*ε* ^2^−*k*) = 0 which is obtained by equating the discriminant in the expression of *x* ^*o*^_±_ to zero.

## Conclusions

In this work we studied the ability of linear augmentation towards stabilizing desired stationary solutions of oscillatory systems in a more general sense. Through some very simple examples discussed in this paper, it is clear that the effectiveness of this scheme is quite sensitive to the augmentation parameters, the class of oscillatory systems considered, the stationary solutions to stabilize and also on the way the systems are augmented. Therefore, although the simplicity of linear augmentation makes it a very compelling choice for applications, a careful analysis is required to test the system for potential pitfalls associated with the scheme. As highlighted by the examples, apart from failing to target the appropriate stationary solutions, linear augmentation can also lead to other complicated dynamical situations which include escaping trajectories, stabilization of unintended stationary solutions or the stabilization of stationary solutions which are not permitted under the modeling constraints; preys existing above their carrying capacities and negative predator populations in Sec. Dissipative predator—prey models for instance. Nevertheless, one can find ways to exploit the failures of the scheme in applications. Although we can expect to see these results in experiments, an in-depth study of the control procedure in presence of noise, and also for larger systems is required. Extending on the results in the ecological context, one needs to check the control behavior in presence of multiple preys and predators, for a food chain, and also for other functional responses [60]. Furthermore, linear augmentation has been proposed as a mechanism to control bistability [34] but how it fares in controlling more general instances of multistability including extreme multistability [47, 61] is still an open question and will be addressed in subsequent studies [60].
